# Self-esteem and existential distress among patients with advanced cancer: mediation by a sense of control and meaning in life

**DOI:** 10.3389/fpsyg.2026.1741247

**Published:** 2026-04-01

**Authors:** Guiru Xu, Guojuan Chen, Huina Zou, Yitao Wei, Huimin Xiao

**Affiliations:** 1 School of Nursing, Fujian Medical University, Fuzhou, China; 2 School of Nursing, Xiamen Medical College, Fujian, China; 3 Nursing Humanities Research Center, Fujian Provincial University Research Base for Humanities and Social Sciences, Fujian, China

**Keywords:** cancer, existential distress, meaning in life, mediating effect, self-esteem, sense of control

## Abstract

**Aim:**

Self-esteem has been reported to be associated with existential distress (ED) in patients with advanced cancer; however, the nature of this association remains insufficiently understood. This study aimed to examine the mechanisms underlying the association between self-esteem and ED in patients with advanced cancer.

**Methods:**

This cross-sectional study was conducted between July 2022 and March 2023. A total of 390 patients with advanced cancer were recruited from two tertiary hospitals in Southeast China using convenience sampling. Self-esteem, sense of control, meaning in life, and ED were assessed. Path analysis was performed to examine the mediating roles of sense of control and meaning in life in the association between self-esteem and ED.

**Result:**

Self-esteem was directly and indirectly associated with ED (*p* < 0.001). Both sense of control and meaning in life significantly mediated the association between self-esteem and ED (*p* < 0.001). The serial mediation pathway through sense of control and meaning in life was also significant and accounted for 21.48% of the total indirect effect (*p* < 0.001).

**Conclusion:**

The findings suggest that self-esteem, sense of control, and meaning in life are interrelated psychological factors associated with ED. Interventions targeting these factors may be relevant in clinical efforts to support patients with advanced cancer experiencing ED.

## 1 Introduction

Patients with advanced cancer frequently endure profound multidimensional suffering as the disease progresses, including severe physical symptoms, functional decline, treatment burden, financial strain, and awareness of a life-limiting prognosis. In this end-of-life context, existential distress (ED) can be understood as a key existential dimension of such suffering, capturing the inner turmoil that emerges when patients confront threats to meaning, dignity, and mortality (Chen et al., 2022; Vehling and Kissane, 2018; Bovero et al., 2018). It reflects individuals’ struggles with meaninglessness, loss of dignity, death anxiety, and perceived loss of control when facing the reality of life and death (Chen et al., 2022; Vehling and Kissane, 2018). Approximately 54–73% of patients with cancer experience ED (Vehling and Kissane, 2018; Bovero et al., 2019), leading to depression, a diminished quality of life, and a potentially heightened risk of suicide (Bovero et al., 2023; Salander, 2018; Huda et al., 2022; Philipp et al., 2025; Xiaodan et al., 2022). If left unaddressed, ED impedes recovery and worsens suffering in patients with cancer (Wang et al., 2025).

Self-esteem is a positive psychological factor that may alleviate ED. It refers to an internal positive psychological attribute that encompasses a positive attitude toward oneself, including one’s values, worth, and reflections (Niveau et al., 2021; Yang et al., 2019). Higher self-esteem is beneficial for dealing with physical and psychological stress. A systematic review by Wang et al. (2023) indicated that self-esteem was negatively related to ED in patients with cancer. However, most of the existing evidence has examined demoralization rather than ED as a distinct construct. Although demoralization overlaps conceptually with ED, the two are not identical in theoretical scope or measurement. Therefore, whether self-esteem is directly associated with ED—particularly among patients with advanced cancer—remains insufficiently verified. Recently, an Italian study indicated that lower self-esteem was associated with higher ED in patients with prostate cancer (Scandurra et al., 2022). It remains unclear whether this finding can be applied to advanced-stage patients with various cancer types and how self-esteem directly affects ED.

Beyond self-esteem, a sense of control is a valuable psychological asset that refers to an individual’s instinctive belief that they can manage their life and environment (Prasad et al., 2023). A low sense of control has been associated with feelings of powerlessness, helplessness, hopelessness, and depression in pathological cases (Hodges and Winstanley, 2012; Anagnosti et al., 2023; Lin and Tsay, 2005), suggesting that a sense of control could be a protective factor against ED. Bovero et al. (2018) indicated that patients with cancer who had lost a sense of control were most vulnerable to developing ED. Similarly, Liu et al. (2025) revealed that a sense of control could negatively affect ED in patients with cancer. Accordingly, increasing the sense of control in patients with advanced cancer may alleviate ED symptoms.

Alongside self-esteem and sense of control, meaning in life refers to an individual’s subjective experience of finding a purpose or direction, understanding life’s circumstances, and experiencing a sense of significance (Winger et al., 2016). Previous studies have shown that meaning in life is negatively associated with distress in cancer populations (Vehling et al., 2011). A meta-analysis of 62 studies further reported a moderate negative correlation between meaning in life and distress among cancer patients (Winger et al., 2016). Longitudinal studies further suggest that meaning functions as a protective factor over time, predicting lower subsequent demoralization, which is closely related to ED (Vehling et al., 2011). Large cohort and multinational studies have consistently linked greater meaning in life with lower levels of existential, or financial distress (Gravier et al., 2020). However, despite this growing body of evidence, relatively few empirical studies have explicitly examined ED as a distinct psychological outcome, particularly among patients with advanced cancer. Moreover, most existing studies have been conducted in Western cultural contexts, and it remains unclear whether these findings can be directly generalized to Chinese patients with advanced cancer. Therefore, it is critical to explore whether meaning in life influences the development of ED in this population.

The interrelationships among self-esteem, sense of control, and meaning in life have been examined in previous studies on patients with cancer and other populations. Rehman (2022) demonstrated that higher self-esteem was positively associated with a stronger sense of control in patients with all stages of breast cancer in Pakistan. Lewis (1982) reported that patients with late-stage cancer with a greater sense of control also had a higher perception of meaningfulness. Furthermore, Liu Zhixiao (2018) found that healthy individuals with higher levels of self-esteem reported greater meaning in life. He also revealed that a sense of control could mediate the relationship between self-esteem and the meaning in life (Liu Zhixiao, 2018). Additional research is required to validate the relationships among self-esteem, sense of control, and meaning in life in patients with advanced cancer.

**Figure 1:**
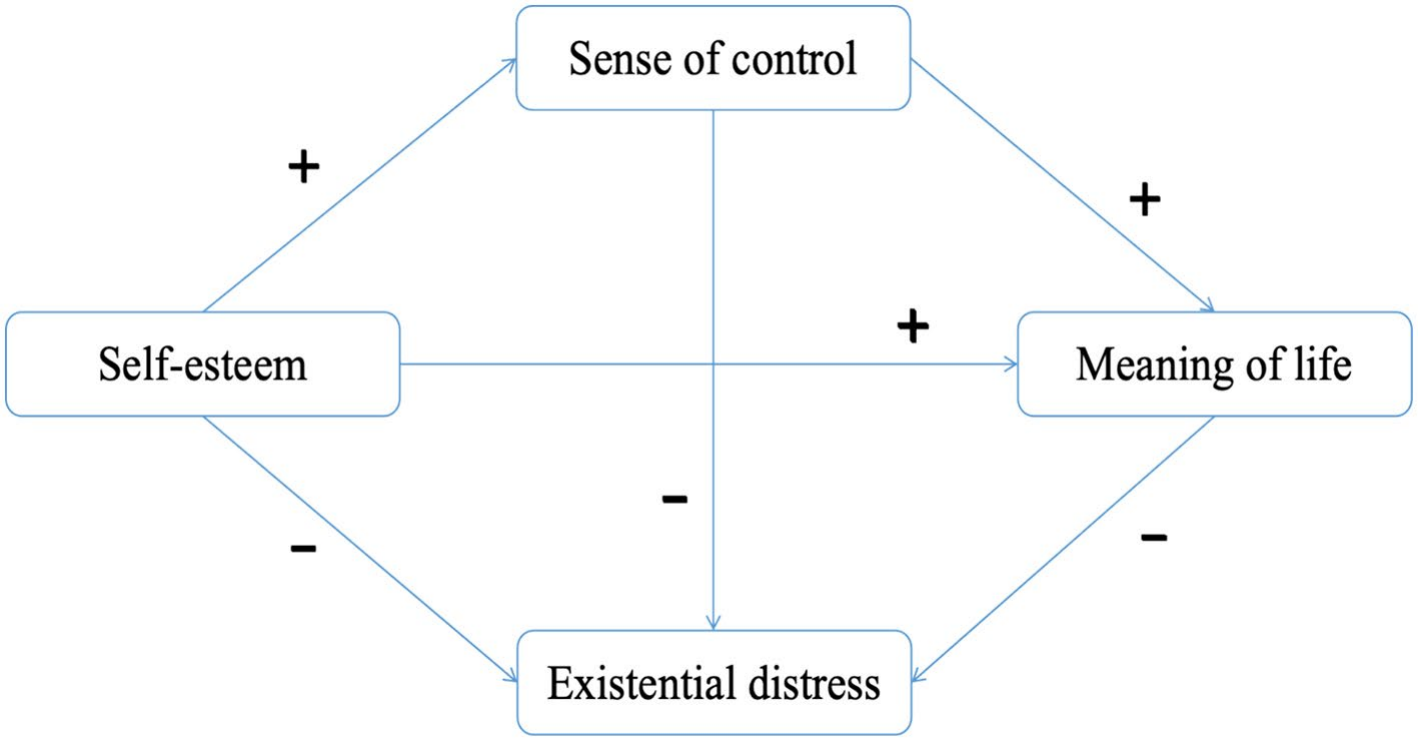
Conceptual model of the study.

Although several studies have reported significant associations among self-esteem, sense of control, meaning in life, and existential distress, there remains a lack of an integrative, theory-driven model that explains how these psychological resources jointly influence ED. Cognitive Adaptation Theory (CAT) developed by psychologists Taylor, it explains that individuals confronted with challenging or traumatic life events can maintain psychological well-being through cognitive processes. The processes involve bolstering one’s self-esteem, retaining a sense of control, and seeking meaning (Taylor, 1983). Based on Cognitive Adaptation Theory and previous empirical findings, the following hypotheses (Figure 1) were proposed:

*H1*: Self-esteem is negatively associated with ED.

*H2*: Sense of control mediates the relationship between self-esteem and ED.

*H3*: Meaning in life mediates the relationship between self-esteem and ED.

*H4*: Self-esteem is positively associated with sense of control.

*H5*: Self-esteem is positively associated with meaning in life.

*H6*: Sense of control is positively associated with meaning in life.

*H7*: Sense of control and meaning in life sequentially mediate the relationship between self-esteem and ED.

This study aimed to test a theory-driven mediation model to clarify how self-esteem influences ED through sense of control and meaning in life in patients with advanced cancer.

## 2 Materials and methods

### 2.1 Design and participants

This cross-sectional study was conducted at two tertiary hospitals in Fujian, China, between July 2022 and March 2023. We conveniently selected participants based on the following criteria: (1) A diagnosis of stage III or IV cancer made by oncologists according to the TNM staging system, and verified by histopathological examination (e.g., biopsy or postoperative pathological report); (2) age of 18 years or older; and (3) awareness of diagnosis and therapy. Participants who were (1) severely disabled or critically ill (Karnofsky Performance Status, KPS < 40); (2) had a visual, hearing, or mental health issue; and (3) experienced a serious adverse event, such as the loss of a loved one, were excluded from the study.

For the descriptive cross-sectional study, the sample size was calculated using the formula *n* = *t*^2^ *p*(1-*p*)/m^2^, where n represents the required sample size, *t* represents the confidence level at 95% (standard value of 1.96), *p* represents the estimated prevalence of ED, and m represents the margin of error at 5% (standard value of 0.05) (Naing et al., 2022). According to a previous study (Nanni et al., 2018), the estimated prevalence of ED is 25%. The calculated sample size was 288; when a 10% non-response rate was considered, the final sample size was 316, which was considered adequate for the path analysis.

### 2.2 Procedure

This study was approved by the corresponding author’s university (No. 20-2018). Two trained research assistants (RAs) were trained to collect the data using Chinese versions of the questionnaires before this study. The training involved the study protocol, eligibility assessment, ethical considerations, questionnaire administration, and communication with patients with advanced cancer. Nurses and doctors in the oncology department initiately screened potential participants with cancer based on the inclusion criteria. Then, the RAs approached eligible patients, explained the study, and obtained written informed consent. They were invited to complete the questionnaires independently. If they experienced difficulty, the RAs read each question aloud verbatim of the Chinese version questionnaires and recorded the responses. All questionnaires were returned on the same day they were distributed. A total of 426 questionnaires were distributed, and data from 390 participants were included in the analysis, resulting in an effective recovery rate of 91.5%. The remaining 36 questionnaires (8.5%) were excluded due to incomplete data (*n* = 12) or over-centralized responses (*n* = 24).

### 2.3 Measures

#### 2.3.1 Demographic and medical characteristics

Demographic information included age, sex, marital status, level of education, religion, monthly household income per capita, and co-residence. A self-reported personal information form was used to collect data. Medical criteria included pain intensity, cancer stage, disease duration (months), surgery (yes or no), chemotherapy (yes or no), and radiation (yes or no). Pain intensity was assessed using the Numeric Rating Scale (NRS; 0–10). Clinical data were obtained from patients’ medical records.

#### 2.3.2 Chinese existential distress scale

The Chinese existential distress scale (CEDS) was used to assess ED (Ying, 2022). CEDS consists of nine items across three dimensions: meaninglessness, estrangement, and death anxiety. Each item is rated on a 4-point Likert scale ranging from 0 (very unlikely) to 4 (extremely likely). The total score, ranging from 0 to 36, was the sum of all item scores, with higher scores indicating higher ED levels. The scale has a Cronbach’s *α* of 0.81, with individual dimensions ranging from 0.70 to 0.76. In the present study, the Cronbach’s α of the scale was 0.72.

#### 2.3.3 Multidimensional health locus of control

The multidimensional health locus of control (MHLC) scale, developed by (Wallston et al., 1994), assesses overall control beliefs regarding disease and health. The MHLC scale consists of 18 items divided into three subscales: Internal Health Locus of Control (IHLC), Powerful Others Health Locus of Control (PHLC), and Chance Health Locus of Control (CHLC). In the present study, only the IHLC subscale was used to assess participants’ sense of control. It includes six items rated on a 6-point Likert scale (1 = strongly disagree to 6 = strongly agree), yielding a total score ranging from 6 to 36, with higher scores indicating a stronger internal sense of control. The Cronbach’s *α* coefficient for the IHLC was 0.68 (Lin and Tsay, 2005). In the present study, the Cronbach’s *α* of the scale was 0.88.

#### 2.3.4 Chinese version of the meaning in life questionnaire

The meaning in life questionnaire (MLQ), developed by Steger et al. (2006), evaluates an individual’s meaning in life. This study used the Chinese version of the MLQ (C-MLQ) adapted by Wang et al. to assess the meaning in life among patients with advanced cancer. The C-MLQ consists of 10 items on a 7-point Likert scale, covering two dimensions: the presence of meaning and search for meaning. Responses range from 1 (“completely inconsistent”) to 7 (“completely consistent”). Higher scores indicate a greater sense of meaning in life. The overall scale’s Cronbach’s *α* was 0.87, with 0.88 for the presence subscale (MLQ-P) and 0.75 for the search subscale (MLQ-S) (Li, 2019). In the present study, the Cronbach’s *α* of the scale was 0.81.

#### 2.3.5 Rosenberg’s self-esteem scale

The Chinese version of Rosenberg’s self-esteem scale (RSES) (Rosenberg, 1965) was used to measure self-esteem in patients with advanced cancer. The scale consists of 10 items, with responses ranging from 1 (strongly disagree) to 4 (strongly agree). The total score ranged from 10 to 40, with higher scores indicating higher self-esteem. The Chinese version of the RSES has good psychometric properties, with a Cronbach’s *α* coefficient of 0.88 (Tian et al., 2021). In the present study, the Cronbach’s *α* of the scale was 0.86.

### 2.4 Data analysis

Data were analyzed using SPSS version 27.0 and AMOS version 24.0. The demographic and medical characteristics of the patients were analyzed using frequency and percentage distributions. Levels of ED, meaning in life, sense of control, and self-esteem were analyzed using means and standard deviations. Spearman’s correlation analysis was performed to explore the correlations between variables because the data did not meet the assumption of normality. The normed *χ*^2^, root mean square error of approximation (RMSEA), incremental fit index (IFI), comparative fit index (CFI), and Tucker–Lewis index (TLI) were used to evaluate the fitness of the hypothetical path model. Normed *χ*^2^ was set to 3 or less; IFI, CFI, and TLI were all greater than 0.90; and RMSEA was set to 0.08 or less (Hair et al., 2014). Bootstrapping was used to confirm the significance of the direct, indirect, and total effects of the variables influencing ED, and 2000 bootstrapping samples were used. Standardized beta coefficients were employed because standardized regression weights could be used to determine the degree of relative influence between endogenous variables. The statistical significance threshold was set at *P* < 0.001.

## 3 Results

### 3.1 Participants’ characteristics

Table 1 presents participants’ characteristics (*n* = 390). Approximately 57.95% of patients with advanced cancer were aged 59 or below, 55.64% were female, approximately three quarters (76.95%) were married, 52.31% held an education level of primary school or below, 79.74% were diagnosed at Stage IV of cancer, 83.08% had undergone or were undergoing chemotherapy, and 55.38% had a disease duration of ≤1 year.

**Table 1:** Characteristics of patients with advanced cancer and existential distress scores (*n* = 390).

Variables	Categories	*N* (%)
Age (years)		
	≤59	226 (57.95)
	≥60	164 (42.05)
Gender		
	Male	173 (44.36)
	Female	217 (55.64)
Marital status		
	Single	5 (1.28)
	Married	298 (76.41)
	Divorced	32 (8.21)
	Widowed	55 (14.1)
Education level		
	Primary school or below	204 (52.31)
	Junior high school	79 (20.26)
	Senior high school	66 (16.92)
	Bachelor’s degree or above	41 (10.51)
Religion		
	Yes	162 (41.54)
	No	228 (58.46)
Monthly household income per capita (CNY)		
	<1000	79 (20.26)
	1,000–3,000	130 (33.33)
	3,001–6,000	110 (28.21)
	>6,000	71 (18.21)
Co-residents		
	Live alone	8 (2.05)
	Spouse	128(32.82)
	Children	47 (12.05)
	Spouse and children	190 (48.72)
	Nanny or home care attendant	5 (1.28)
	Others	12 (3.08)
Diagnosis		
	Digestive system cancers	180 (46.15)
	Respiratory system cancers	127 (32.56)
	Other cancers	83 (21.29)
Pain		
	No	83 (12.8)
	Mild	224 (57.44)
	Moderate	71 (18.21)
	Severe	12 (3.07)
Stage of cancer		
	III	79 (20.30)
	IV	311 (79.70)
Disease duration (month)		
	1–12	216 (55.38)
	13–24	70 (17.95)
	25–36	43 (11.03)
	≥37	61 (15.64)
Surgery		
	Yes	186 (47.69)
	No	204 (52.31)
Chemotherapy		
	Yes	324 (83.08)
	No	66 (16.92)
Radiotherapy		
	Yes	76 (19.49)
	No	314 (80.51)

### 3.2 Descriptive statistics and correlations of variables

Table 2 shows the ED, self-esteem, sense of control, and meaning in life scores and their relationships. The scores of ED, self-esteem, sense of control, and meaning in life were 13.80 ± 4.58, 28.05 ± 4.60, 16.64 ± 5.85, and 39. 50 ± 5.54, respectively. ED was negatively correlated with self-esteem (*r* = −0.667, *p* < 0.001), sense of control (*r* = −0.569, *p* < 0.001), and meaning in life (*r* = −0.754, *p* < 0.001).

**Table 2:** Correlation relationship and mean scores among CEDS, RSES, IHLC, and C-MLQ (*n* = 390).

Variables	*M* ± SD	Correlations among variables			
		ED	Self-esteem	Sense of control	Meaning in life
ED	13.80 ± 4.58	1			
Self-esteem	28.05 ± 4.60	−0.667^**^			
Sense of control	16.64 ± 5.85	−0.569^**^	0.359^**^		
Meaning in life	39.50 ± 5.54	−0.754^**^	0.547^**^	0.494^**^	1

** Indicates statistical significance at *p* < 0.001.

### 3.3 Mediating effects of sense of control and meaning in life

**Figure 2:**
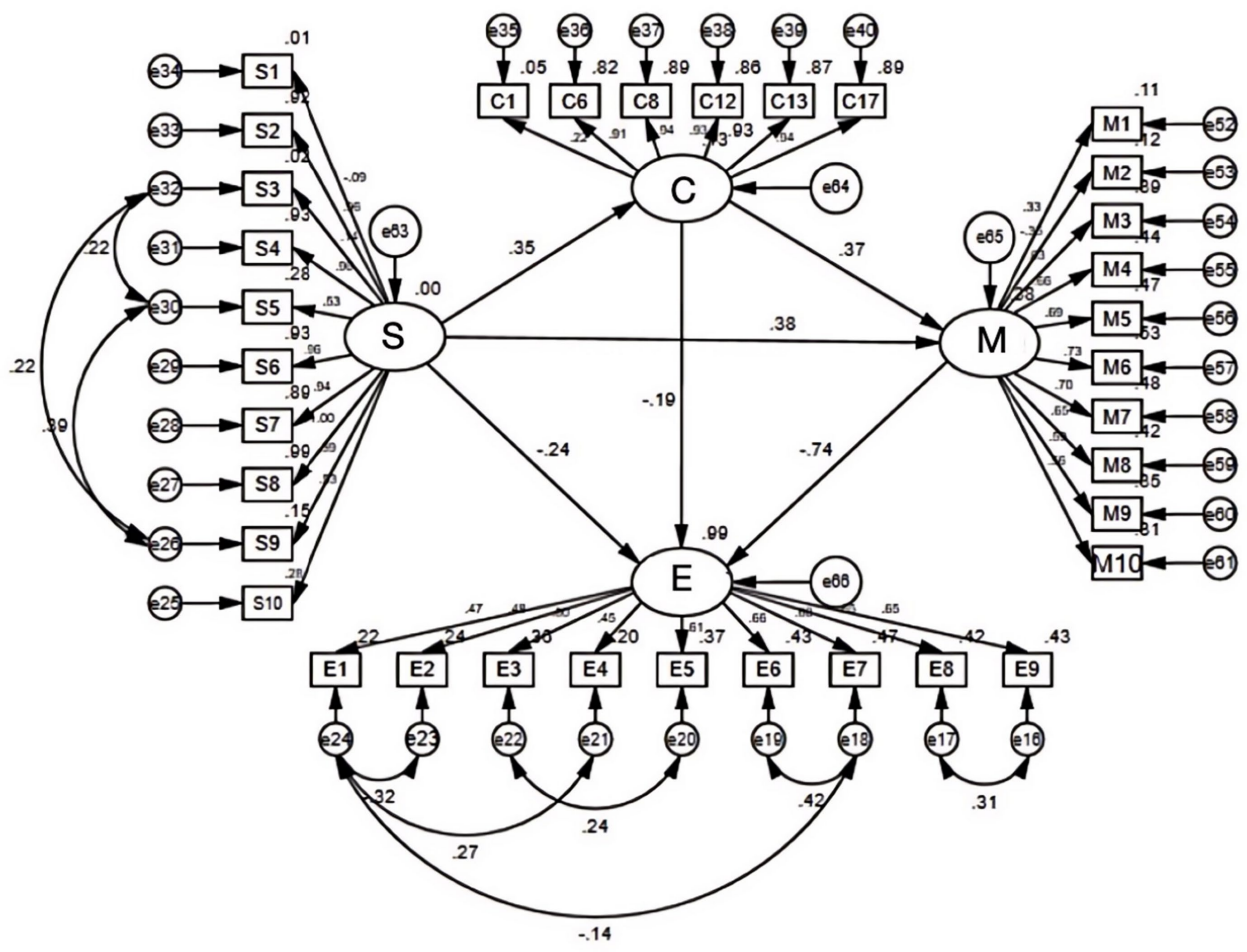
Results of the final path model (*n* = 390). Standardized coefficients are shown in the figure. S, self-esteem; C, sense of control; M, meaning in life; E, existential distress.

Table 3 presents the findings regarding the mediating effects of sense of control and meaning in life on the relationship between self-esteem and ED, and Figure 2 shows the final path model. All path coefficients reported in this study are standardized regression coefficients. The goodness of fit in the hypothetical path model was *X*^2^/df = 2.156, RMSEA = 0.055, TLI = 0.933, CFI = 0.939, and IFI = 0.939, indicating a good overall model fit. The total effect of self-esteem on ED was significant (*b* = −0.685, SE = 0.037, *p* = 0.001, percentile CI = [−0.757, −0.615]). The direct effect accounted for 34.74% of the total effect (*b* = −0.238, SE = 0.042, *p* = 0.001, percentile CI = [−0.327, −0.162]), and the mediating effect accounted for 65.26% of the total effect (*b* = −0.447, SE = 0.047, *p* < 0.001, percentile CI = [−0.540, −0.359]). Within the mediating effect, both sense of control and meaning in life significantly mediated the relationship between self-esteem and ED (*b* = −0.068, SE = 0.013, *p* = 0.001, percentile CI = [−0.077, −0.026]; *b* = −0.283, SE = 0.400, *p* < 0.001, percentile CI = [−0.303, −0.143]), and accounted for 15.21 and 63.31% of the mediating effect, respectively. The chain mediation of sense of control on meaning in life was also significant and accounted for 21.48% of the total mediation effect (*b* = −0.096, SE = 0.017, *p* < 0.001, percentile CI = [−0.112, −0.045]).

**Table 3:** Mediating effects of a sense of control and meaning in life on the relationships between self-esteem and ED (*n* = 390).

Paths	Point estimate	SE	*p*	Percentile CI	
				Lower	Upper
Total effects Self-esteem → ED	−0.685	0.037	0.001	−0.757	−0.615
Direct effects Self-esteem → ED	−0.238	0.042	0.001	−0.327	−0.162
Indirect effects Total indirect effects	−0.447	0.047	<0.001	−0.540	−0.359
Self-esteem → a sense of control → ED	−0.068	0.013	0.001	−0.077	−0.026
Self-esteem → meaning in life → ED	−0.283	0.400	<0.001	−0.303	−0.143
Self-esteem → a sense of control → meaning in life → ED	−0.096	0.017	<0.001	−0.112	−0.045

## 4 Discussion

This study provides new evidence regarding the relationships among self-esteem, sense of control, meaning in life, and ED in patients with advanced cancer. The findings help elucidate the patterns of association through which self-esteem is related to ED. The structural model indicated that self-esteem, sense of control, and meaning in life were significantly associated with ED in this population. Furthermore, sense of control and meaning in life statistically mediated the association between self-esteem and ED, both individually and sequentially.

As predicted, self-esteem was inversely associated with ED, with the direct pathway accounting for 34.74% of the total association between self-esteem and ED among Chinese patients with advanced cancer. This finding is consistent with those of similar studies. According to stress and coping theories, self-esteem is conceptualized as a personal resource that may be linked to better psychological adjustment under severe stress, such as advanced cancer (Taylor, 1983). Scholars suggest that people with higher self-esteem may feel more confident in their capacity to face illness-related challenges, which may be associated with lower feelings of worthlessness and hopelessness (Yang et al., 2019; Yu et al., 2021). Furthermore, earlier research conducted in Taiwan (Li et al., 2015) and Indonesia (Aprilianto et al., 2021) found that patients with cancer with better self-esteem tended to report stronger ties with family and friends and larger social support networks. Such social support has been associated with lower levels of estrangement and loneliness, which are key components of ED.

Consistent with prior research, individuals with higher self-esteem tend to report a stronger sense of control (Náfrádi et al., 2017). Our findings indicate that sense of control was associated with the relationship between self-esteem and ED. This pattern may reflect that individuals with higher self-esteem perceive themselves as more capable of influencing aspects of their illness experience. Such perceptions may be associated with greater confidence in handling the complexities of cancer treatment and care, which may correspond to a stronger sense of control (Ros-Sanchez et al., 2023; Ruiz-Romeo et al., 2025). Furthermore, Idris et al. (2023) and Özkan et al. (2023) reported that individuals with higher self-esteem were more likely to play an active role in healthcare decisions. By gathering information, asking questions, and engaging in treatment planning, individuals may report a greater sense of agency and control over their health and treatment.

In contrast, a higher sense of control was negatively associated with ED. This association may be linked to lower levels of self-perceived burden (Rothman et al., 2023) and to fewer negative psychological states, such as depression and anxiety (Ferreira Couto and Nunes Baptista, 2023; Dağ and Şen, 2018), which are key attributes of ED. Additionally, patients who believe they have greater control over their condition may report fewer feelings of powerlessness, hopelessness, fear of the future, and death (Nwonyi et al., 2024; Cao et al., 2023), which are the core attributes of the ED. Therefore, in this sample, patients with advanced cancer who reported a stronger sense of control also tended to report lower ED.

The current study also found that meaning in life statistically mediated the association between self-esteem and ED. Higher self-esteem was associated with greater meaning in life, which in turn was associated with lower levels of ED. The association between self-esteem and meaning in life may be interpreted in light of the meaning maintenance model (MMM), which conceptualizes self-esteem as an important factor related to meaning in life (Heine et al., 2006). Increased self-esteem is frequently related to more positive social relationships, which may partly account for the association between self-esteem and meaning in life in our results (Zulkarnain and Vanoh, 2024). As Stavrova and Luhmann (2016) showed, strong and supportive relationships are associated with a greater sense of belonging and significance, thereby corresponding to higher meaning in life.

Furthermore, patients with higher self-esteem have been reported to be more optimistic and hopeful (Montañés-Muro et al., 2023; Dargan et al., 2021). This positive orientation may be associated with greater perceived significance in one’s experiences, including framing illness-related experiences within a broader and more meaningful life narrative. In turn, higher meaning in life has been associated with lower levels of ED. In Quinto’s review (Quinto et al., 2022), patients with higher meaning in life were described as being more accepting of cancer and the end-of-life context, which was associated with less resistance and distress when facing mortality. Therefore, patients with advanced cancer who report higher self-esteem may also report higher meaning in life, which is associated with lower ED.

According to our findings, the serial mediation effects of sense of control and meaning in life on ED accounted for 21.48% of the total indirect effects. This suggests that self-esteem may be related to ED through its associations with sense of control and meaning in life. Patients with a stronger sense of control may be more active in developing and pursuing personal goals, even in the midst of illness. Attaining objectives, regardless of how small, may be associated with a sense of success and purpose and may be linked to higher meaning in life (Machell et al., 2015). In our study, patients with advanced cancer and a stronger sense of control were more likely to report striving for daily and health goals, attributing outcomes to personal efforts, and actively seeking meaning in their lives. Thus, sense of control was positively associated with meaning in life, which in turn was linked to lower levels of ED. This highlights the potential relevance of control- and meaning-related resources when considering approaches to address ED in patients with advanced cancer.

### 4.1 Limitations

This study has certain limitations. The cross-sectional design prevented us from making causal inferences regarding self-esteem, sense of control, meaning in life, and ED. Also, convenience sampling may restrict the generalisability of the results. Finally, relying solely on self-reported measures could introduce a desirability bias, potentially affecting how the study’s findings are interpreted. To enhance the credibility of future research, employing a longitudinal or experimental approach with mixed methods, including interviews, could provide additional validation of the findings.

### 4.2 Relevance to clinical practice

Given the life-threatening nature of advanced cancer, addressing palliative care and enhancing patients’ existential well-being are crucial for healthcare providers. This study offers insights into how self-esteem, sense of control, and meaning in life are associated with ED. Accordingly, clinical efforts that aim to support these psychological resources may be relevant for patients experiencing ED. For example, clinicians could encourage patients to reflect on positive life experiences in ways that may support self-esteem, provide skills-based or cognitive strategies that may enhance perceived control, and implement meaning-focused approaches that may strengthen patients’ perceived meaning in life. In addition, interventions that facilitate social connectedness and shared decision-making may be associated with higher self-value and agency, which are relevant correlates of ED.

## 5 Conclusion

To the best of our knowledge, this is the first study to examine the mediating roles of sense of control and meaning in life in the association between self-esteem and ED. Self-esteem was directly associated with ED and indirectly associated with ED through three pathways: (1) the mediating role of sense of control; (2) the mediating role of meaning in life; and (3) a serial mediation pathway involving both sense of control and meaning in life. These findings suggest that self-esteem, sense of control, and meaning in life are interrelated psychological resources that are associated with ED. Accordingly, comprehensive clinical approaches that support these positive psychological factors may be relevant when addressing ED in patients with advanced cancer.

## Data Availability

The raw data supporting the conclusions of this article will be made available by the authors, without undue reservation.
